# Elevated Levels of IL–1Ra, IL–1β, and Oxidative Stress in COVID-19: Implications for Inflammatory Pathogenesis

**DOI:** 10.3390/jcm14072489

**Published:** 2025-04-05

**Authors:** Alicja Marczewska, Celina Wojciechowska, Kamil Marczewski, Natalia Gospodarczyk, Paweł Dolibog, Zenon Czuba, Karolina Wróbel, Jolanta Zalejska-Fiolka

**Affiliations:** 1 Department of Biochemistry, Faculty of Medical Sciences in Zabrze, Medical University of Silesia, 41-808 Zabrze, Poland; 2 Second Department of Cardiology, Faculty of Medical Sciences in Zabrze, Medical University of Silesia, 41-808 Zabrze, Poland; 3 Department of Urology, Faculty of Medical Sciences in Katowice, Medical University of Silesia, Plac Medyków 1, 41-200 Sosnowiec, Poland; 4 Department of Ophthalmology, Faculty of Medical Sciences in Zabrze, Medical University of Silesia in Katowice, 40-055 Katowice, Poland; 5 Department of Biophysics, Faculty of Medical Sciences, Medical University of Silesia, 41-808 Zabrze, Poland; 6 Department of Microbiology and Immunology, Medical University of Silesia in Katowice, 41-808 Zabrze, Poland; 7 Medical Laboratory of Teresa Fryda Katowice, Katowice, Laboratory Branch in Specialist Hospital in Zabrze, 10, M.C-Skłodowska St., 41-800 Zabrze, Poland

**Keywords:** COVID-19, cytokines, oxidative stress, SARS-CoV-2 virus

## Abstract

**Background:** The coronavirus–caused disease (COVID-19), first identified in China in December 2019, has spread worldwide becoming a global pandemic. Although people infected with the SARS-CoV-2 virus presented mainly respiratory and gastrointestinal symptoms, an increase in cardiovascular incidents was observed in several scientific studies. SARS-CoV-2 virus has been shown to disrupt the normal immune response leading to a dysregulation of immune system function and massive production of inflammatory cytokines commonly known as “cytokine storm”. **Methods:** 57 patients eventually participated in the study, assigned to non–COVID (24 patients) and COVID (33 patients) groups. After signing consent to participate in the study, each patient was given a self–administered questionnaire to fill out prior to specimen collection, anthropometric measurements were taken and venous blood was collected for the following determinations: pro- and anti–inflammatory cytokines using a Bio–Plex 200 system, oxidative stress markers and basic hematological blood parameters. **Results:** showed statistically significant higher values of IL–1Ra and IL–1β in the COVID-19 group. Of the oxidative stress markers, only MDA levels were higher in the COVID-19 group. **Conclusions:** the results of our study provide evidence and support the occurrence of elevated levels of IL–1Ra, IL–1β and MDA in the COVID-19 group of patients, which are associated with a worse course and prognosis of COVID-19. A better understanding of the pathophysiology and dysregulation of the immune system associated with the cytokine storm is essential to select patients at risk and develop effective drugs and vaccines.

## 1. Introduction

The coronavirus-associated disease (COVID-19), first identified in China in the city of Wuhan in December 2019, has spread worldwide, becoming a global pandemic with high morbidity and mortality and huge social, economic, and cultural impact [1]. According to WHO data, as of today (8 January 2025), the severe acute respiratory syndrome–CoV–2 (SARS-CoV-2) virus has become the cause of more than 777,112,363 confirmed cases of infection and 7,079,587 deaths worldwide, with almost 6.8 million cases and more than 121,000 deaths in Poland [2]. Although people infected with SARS-CoV-2 presented mainly with respiratory and gastrointestinal symptoms, an increase in the incidence of cardiovascular incidents in the form of atherosclerosis and thromboembolic complications has been observed in several scientific studies [3,4]. Atherosclerosis is a process resulting from local lipid deposition in the blood vessel wall, leading to vessel narrowing. It can be caused by various factors such as smoking, a diet high in saturated fat, hyperglycemia, hypertension, and genetic factors [5,6]. There is ample evidence in the available literature that atherosclerosis can develop due to viral and bacterial infection [7,8,9]. SARS-CoV-2 virus has been shown to disrupt the normal immune response, leading to the dysregulation of immune function and the massive production of inflammatory cytokines, commonly known as a “cytokine storm”. In response to infection, activated neutrophils and macrophages produce massive amounts of cytokines, toxic compounds, enzymes, and reactive oxygen species. Increased inflammation results in the development of oxidative stress, which stimulates further cytokine release, triggering a vicious cycle of self-perpetuating inflammation and ultimately the development of a cytokine storm. A number of reports have suggested that the severe course and complications of COVID-19 are not due to the direct effects of SARS-CoV-2 but are due to excessive cytokine release in response to viral infection. In the case of SARS-CoV-2 infection, IL–1, IL–6, and TNF–α play a major role in this process [10].

The aim of the present study was to quantify the expression levels of the interleukin-1 receptor antagonist (IL–1Ra), the inflammatory cytokine IL–1β, oxidative stress markers, and baseline blood hematological parameters among patients with COVID-19 compared with a group of patients without SARS-CoV-2 infection. Although several papers have described mechanisms and processes that explain with high probability the correlation between the course of COVID-19 and the occurrence of complications, in the form of thromboembolic incidents, the situation regarding SARS-CoV-2 infection is changing dynamically. Therefore, further studies are needed to thoroughly understand the hemodynamic mechanisms and the cardiovascular impact of the infection, which will allow for the proper selection and choice of the appropriate therapy in COVID-19 patients. The results of our study provide further evidence and support the presence of elevated levels of IL–1Ra, IL–1β, and malondialdehyde (MDA) in COVID-19 patients, which are associated with a worse course and prognosis of COVID-19. A better understanding of the pathophysiology and dysregulation of the immune system associated with the cytokine storm is essential to select patients at risk and to develop effective drugs and vaccines. In addition, knowledge of the mechanism of SARS-CoV-2 infection will enable the development and introduction of predictive diagnostic tests to predict the course and severity of the disease.

## 2. Material and Methods

### 2.1. Patients

This study was carried out between 2021 and 2024 in the Department of Biochemistry and the Medical Laboratory of Dr Teresa Fryda, n.d., in Zabrze. A total of 117 subjects participated in the study, from which a group of 94 patients was selected after taking into account the exclusion criteria. Due to the need for group homogeneity and the small number of unvaccinated subjects (8), it was decided to exclude subjects who declared that they had not received any COVID-19 vaccine doses. Due to the small number of unvaccinated subjects, statistics were compiled only within the vaccinated subjects (86). Vaccinated patients were divided into two groups:

1. Those with a confirmed history of SARS-CoV-2 infection (COVID) (n = 33).
2. Control group: Subjects who declared that they had not experienced COVID-19 (non-COVID) (n = 24).

Those who marked the answer “don’t know” (n = 29) were not included in the statistical analysis due to inability to classify these patients clearly.

**Figure 1:**
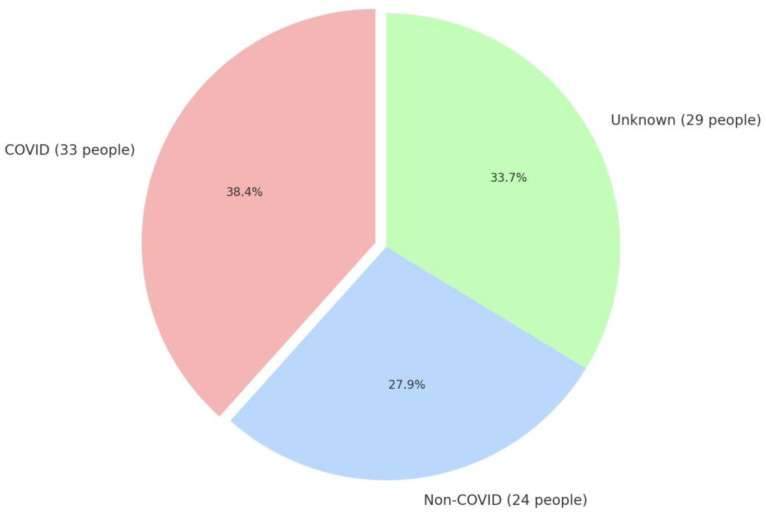
Distribution of vaccinated patients in the study.

Below Figure 1 shows the distribution of vaccinated patients in the study.

### 2.2. Procedure

Patients who reported for examinations at the Diagnostyka medical laboratory in Zabrze were included in the study, including 40 PLUS preventive examinations. After signing their consent to participate in the study, each patient was given a self-administered questionnaire to complete before the material was collected, concerning current medical conditions, medications and stimulants taken, family history, and information on past SARS-CoV-2 infection and COVID-19 vaccination. Anthropometric measurements were taken, and an additional 10 mL of venous blood was collected. The collected blood was centrifuged to obtain serum, which was stored at -80 degrees Celsius until biochemical determinations were made. To avoid bias, all samples have been anonymized and numbered. Patients’ data are protected by a RODO statement. This study was approved by the Bioethical Committee of the Medical University of Silesia (SUM), with the reference number PCN/CBN/0022/KB1/134/21 (21 December 2021).

Inclusion criteria were informed consent to participate in the study, no therapy with drugs affecting lipid metabolism and coagulation, and age > 18 years. Exclusion criteria included patients with a history of coronary artery disease, arteriosclerosis of the lower extremities or carotid arteries, BMI > 40, severe liver failure, renal failure, severe respiratory failure, circulatory failure, cancer on therapy, impaired consciousness, psychiatric illness, alcohol and substance abuse, and pregnancy.

### 2.3. Data Analysis

All procedures followed good laboratory practice (GLP). The biochemical analyses were carried out in blinded samples by experienced scientific and technical staff of the Department of Microbiology and Biochemistry and Immunology, Silesian Medical University in Zabrze. Biochemical analyses were performed using Bio-Rad’s Bio-Plex Pro Human Cytokine 27-Plex Panel system (M500KCAF0Y). The representative working range and sensitivity of the assay were as follows: specificity, analyte cross-reactivity <10%, intra-assay precision % CV (coefficient of variation) < 15%, accuracy, and percentage recovery 70–130%. The Bio-Plex Suspension Array system had fluorescently labeled microspheres and instrumentation licensed from Bio-Rad Laboratories, Inc., by the Luminex Corporation (Austin, TX, USA). Calibration curves were produced using reference standards supplied with the kits and used to determine the appropriate analyte concentrations for each sample.

### 2.4. Statistical Analysis

All statistical analyses were performed using the STATISTICA software v 13.3 (TIBCO Software Inc., Palo Alto, CA, USA, 2017). The normality of data distribution was tested by means of the Shapiro–Wilk test. For comparative analysis, the non-parametric Mann–Whitney U test was applied to variables with non-parametric distribution. The chi-square test was used to examine differences between groups for qualitative data. Statistical significance was assumed at a *p*-value of <0.05.

## 3. Results

The final study included 57 patients allocated to the non–COVID (13 women and 11 men) and COVID (15 women and 18 men) groups. Patients’ demographics are shown in Table 1.

In the non–COVID group, the median age was 48.5 years (range: 41.0–75.0); while in the COVID group, it was 49.0 years (range: 35.0–86.0). In contrast, the median BMI in the non–COVID group was 26.03 kg/m^2^; while in the COVID group, it was 26.09 kg/m^2^ (Table 1). The groups were homogeneous in terms of the number of patients, gender, smoking, obesity, physical activity, age, height, and weight.

Of the parameters studied, triglyceride levels were significantly lower in the COVID group compared to the non–COVID group (Table 2); moreover, creatinine levels were significantly higher in the COVID group compared to the non–COVID group (Table 2). Regarding AST, GGTP, cholesterol, uric acid, and glucose levels, there were no significant statistical differences between the study groups.

**Figure 2:**
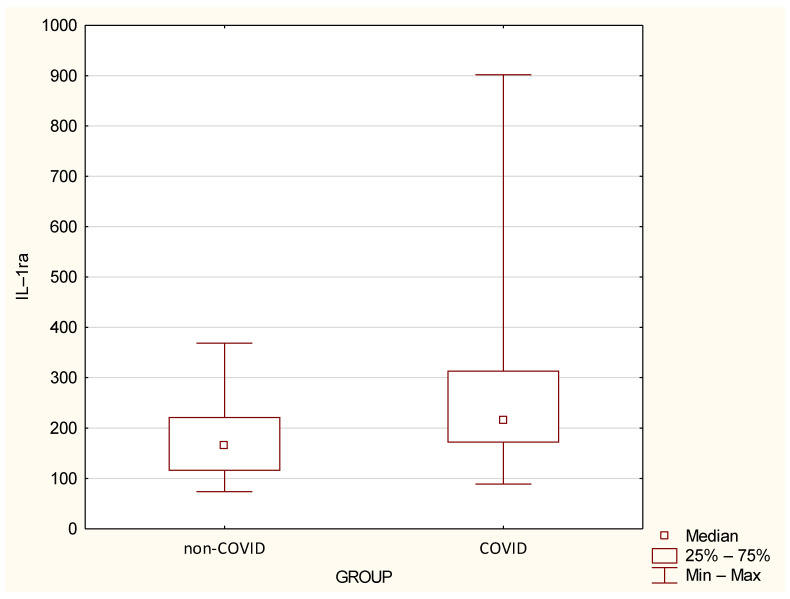
Serum IL–1Ra levels in the study groups. Boxes represent the first and third quartile ranges; whiskers represent minimum and maximum values. Differences between groups are statistically significant at *p* < 0.05.

**Figure 3:**
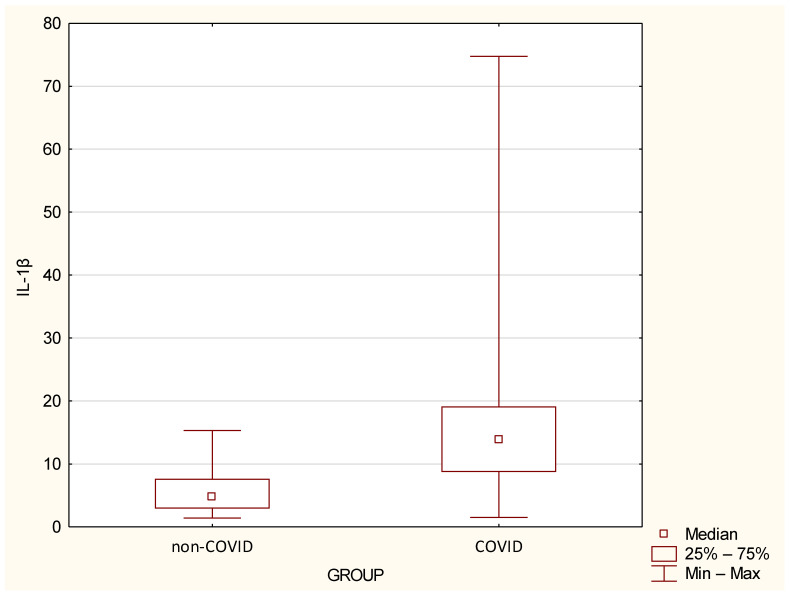
Serum IL–1β levels in the study groups. Boxes represent the first and third quartile ranges; whiskers represent minimum and maximum values. Differences between groups are statistically significant at *p* < 0.05.

Cytokine levels in the non–COVID and COVID groups are detailed in Figure 2 and Figure 3 and Table 3. The COVID group had a median IL–1Ra of 214.72 (range: 88.77–901.91); while the non–COVID group had a median of 166.09 (range: 73.95–368.64). IL–1Ra levels were significantly higher in the COVID group compared to the non-COVID group (*p* < 0.05). The COVID group had a median IL–1β of 13.88 (range: 1.51–74.76); while the non–COVID group had a median of 4.79 (range: 1.42–15.35). Furthermore, the inflammatory cytokine IL–1β showed statistically significantly higher values in the COVID group compared to the non–COVID group (*p* < 0.05).

**Figure 4:**
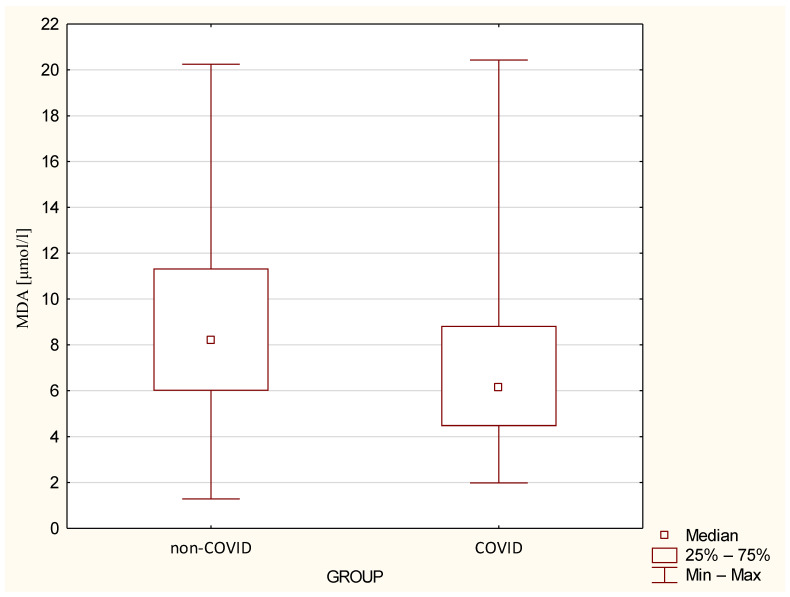
Serum MDA levels [μmol/L] in the study groups. The boxes represent the first and third quartile ranges; whiskers represent minimum and maximum values. Differences between groups are statistically significant at *p* < 0.05.

Only MDA levels in the COVID group were significantly higher than in the non–COVID group (Figure 4, *p* = 0.037, respectively), with a median in the COVID group of 6.15 μmol/L (range: 1.98–20.43) and a median in the non–COVID group of 8.2 μmol/L (range: 1.28–20.25). There were no differences in levels of CER, SH, TOS, LPH, TAC, SOD, MnSOD, CuZnSOD, LPS, and AGE10 MCP-1 and MIP-1 between the groups (Table 4).

## 4. Discussion

In the present study, we quantified the expression levels of IL–1Ra, the cytokine IL–1β, the oxidative stress markers, and the baseline blood hematological parameters among patients with COVID-19 compared with a group of patients without SARS-CoV-2 infection. We found statistically significantly higher values of IL–1Ra and IL–1β in the COVID-19 group. Of the oxidative stress markers, only MDA levels were higher in the COVID-19 group.

Cytokines are proteins produced by immune cells that regulate the body’s immune response. They act as mediators of inflammation, affecting the growth, proliferation, and differentiation of other cells [11]. In some infectious diseases, excessive inflammation leads to the activation of a “cytokine storm”, which causes a range of pathological changes and even multi-organ dysfunction. A number of previous studies have shown that the cytokine storm is associated with the severity of influenza, SARS, and MERS, and more recent studies also confirm its association with SARS-CoV-2 infection [12,13,14].

One of the main mediators of inflammation is interleukin 1 (IL–1), which, through its IL–1 type 1 receptor (IL–1R1), leads to autoimmunity and the development of a number of autoinflammatory diseases [15]. The IL–1 signaling pathway is initiated by the binding of interleukin 1 alpha (IL–1α) or interleukin 1 beta (IL–1β) to the IL–1R1 receptor. This leads to the formation of the receptor complex and the activation of the nuclear factor kappa B (NF–κB) and mitogen-activated protein kinase (MAPK) signaling pathways, causing further expression of pro-inflammatory cytokines and chemokines. The IL–1 receptor antagonist (IL–1Ra) competes with IL–1α and IL–1β for IL–1R1 binding, acting as an inhibitor of the IL–1 signaling cascade. IL–1Ra deficiency has been shown to lead to the development of multifocal inflammation: skin inflammation, bone inflammation, development of interstitial lung disease, CNS vasculitis, and respiratory failure [16,17].

The respiratory system is equipped with a specialized immune system that protects the lungs from damage associated with the influx of pathogens from the external environment. Regulatory T lymphocytes (Treg) suppress the immune response, prevent autoimmunity, and regulate inflammatory processes. They have a particular role in lung-related diseases, where they suppress the excessive immune response, preventing the development of acute and chronic inflammation. Tregs exhibit immunosuppressive effects by, among other things, secreting anti-inflammatory cytokines and thereby preventing tissue damage. In addition, they act as a receptor for interleukin 33 (IL–33), which mediates tissue repair in response to IL–33 [18,19]. In a study by Griffith et al., it was shown that deletion of the receptor for IL–33 on Treg cells led to an increased influx of neutrophils into the lungs and increased cytokine production. After pathogen exposure, IL–33–activated Treg cells led to increased production of IL–1Ra, which, by blocking the action of IL–1, inhibited chemokine secretion by lung fibroblasts, which was beneficial in reducing inflammation. Thus, the immunosuppressive effect of IL–1Ra, secreted by Treg, on respiratory viral infection mediated by IL–1 has been described [20,21].

With the proven impact of IL–1 glut in the development of inflammatory diseases, it became important to look for anti-inflammatory therapies targeting IL–1, preferably without affecting IL–1Ra. The first biologic drug introduced for the treatment of certain inflammatory diseases was anakinra, a selective IL–1R1 antagonist administered by daily injection [19]. Another approved agent for anti-inflammatory therapy was canakinumab, a monoclonal antibody directed against IL–1β but, unfortunately, not against IL–1α [22]. US Food and Drug Administration (FDA) approval was also granted to rilonacept, which is used to treat IL–1Ra deficiency and recurrent pericarditis. This drug blocked IL–1 signaling by acting as a receptor binding site for IL–1α, IL–1β, and IL–1Ra. However, its efficacy was not satisfactory due to the mass neutralization of IL–1Ra, whose deficiency caused inflammation [19,23,24].

Despite the introduction of the above anti-IL–1 drugs, there is a continued need to search for other effective anti-inflammatory therapies due to their inadequacies. The observed clinical and laboratory improvements associated with the use of inhibitors of the IL–1 pathway are hopeful.

In our study, elevated IL–1 levels in COVID-19 patients were indicative of an ongoing inflammatory process. In response, compensatory mechanisms were activated to inhibit the IL–1 signaling pathway. One compensatory mechanism was the increased secretion of IL–1Ra, the levels of which were significantly higher in patients with SARS-CoV-2 infection. Similar to our study, elevated levels of IL–1Ra have been detected in inflammatory, IL–1-dependent diseases such as pyrine-associated autoinflammation with neutrophilic dermatosis (PAAND) and neonatal inflammatory multisystem disease (NOMID), presumably to balance inflammation [25]. The results of our study may also shed light on the usefulness of anti-IL–1 therapy in the treatment of inflammatory diseases other than COVID-19.

A study by Liu et al. assessed the effect of aspirin on the severity of COVID-19 and the association between IL–1Ra and COVID-19. It was shown that aspirin treatment was associated with a lower severity of SARS-CoV-2 infection; moreover, aspirin administration led to a decrease in serum IL–1Ra levels [26,27]. The action of aspirin, as a drug of the non-steroidal anti-inflammatory group, is to inhibit cyclooxygenase and reduce prostaglandin production, thereby affecting the inflammatory process [28]. IL–1Ra also shows an anti-inflammatory effect due to its inhibition of the IL–1 pathway. It is expressed in inflammatory cells such as macrophages, monocytes, and dendritic cells, and its levels increase in acute inflammation [29]. Higher levels of cytokines, including IL–1Ra, were found in patients with severe COVID-19. This confirms that during the acute phase of the disease, inflammatory cells are activated and secrete excessive cytokines, ultimately leading to the development of the so-called “cytokine storm”. It has been shown that clinical deterioration and death in patients with a severe course was associated with increased IL–1Ra expression, indicating a link between the severe course of infection and the cytokine storm [26,30]. In addition, it was observed that under the influence of aspirin, there was a reduced expression of IL–1Ra and thus a milder course of infection, probably due to the inhibitory effect of aspirin on the inflammatory response [26]. The above study provided evidence that aspirin may improve the course of COVID-19, but this mechanism requires further investigation.

Another study by Jacobs et al. showed that 24 months after SARS-CoV-2 virus infection, patients still presented some of their symptoms, which correlated with the levels of IL–1Ra and the endothelial dysfunction markers determined (mainly endothelin–1) [31]. Two years after infection, IL–1Ra levels were positively correlated with the presence of symptoms such as fatigue and sleep or mobility problems. Thus, the pathogenesis of the presented COVID-19 symptoms was confirmed to be related to systemic inflammation and microvascular dysfunction [31]. These data suggest that anti-inflammatory therapies may be beneficial for COVID-19 symptomatic patients even several months after SARS-CoV-2 infection.

A similar problem was assessed in a large study that recruited 8077 people in Germany. Of the first 318 people, almost 70% were found to have the following symptoms, persisting for a period of about eight months after SARS-CoV-2 infection: fatigue, shortness of breath, and impaired concentration. These symptoms were associated with elevated levels of pro-inflammatory cytokines: IL–1β, IL–6, and TNF. In the first phase of the study, participants in the acute phase of COVID-19 presented patterns of elevated inflammation in the form of high concentrations of cytokines such as TNF (TNF–α), LTA (TNF–β), IL–1β, IL–4, IL–6, IL–8, IL–13, and interferon (IFN) –α2. Of these, only three—IL–1β, IL–6, and TNF—revealed a significant correlation with persistent clinical symptoms after a long follow-up of 8 months. The data provided by Schultheiß et al. therefore suggest that persistently high levels of IL–1β, IL–6, and TNF could potentially be one of the pathomechanisms of many COVID-19 symptoms [32]. As 20% of the participants reported resolution of symptoms 4–12 weeks after infection, the trends of IL–1β, IL–6, and TNF levels in these patients over time were also assessed. However, there was no clear decrease in the serum levels of these cytokines in symptom-free patients. As the levels of most cytokines decrease after SARS-CoV-2 infection, whereas the levels of IL–1β, IL–6,and TNF are stable over a longer period of time, it was assessed what mechanisms are involved in this process [32]. This triad of cytokines has been shown to be specifically secreted by pulmonary monocytes and macrophages, as confirmed in previous publications analysing single cells of lung tissue, bronchoalveolar fluid and serum from patients with acute COVID-19 [33,34]. For the above reasons, IL–1β, IL–6, and TNF can be readily interpreted as mediators of the ongoing immune response induced by SARS-CoV-2 infection. Furthermore, the long-term persistence of high plasma levels of IL–1β, IL–6, and TNF in patients with COVID-19 symptoms opens up therapeutic options based on blocking the signaling of these cytokines [32].

Based on the available studies, no reduced levels of IL–1Ra and IL–1β were found in patients with COVID-19. In contrast, an experiment was performed in which the effects of exercise on thermoregulatory, cardiorespiratory responses, and levels of IL–1Ra and IL–6 were assessed in patients with a history of SARS-CoV-2 infection. No significant differences in IL–Ra levels were found between the COVID-19 patient group and a control group without a history of infection [35].

In addition to inflammation, the evaluation of other pathophysiological mechanisms, including the contribution of oxidative stress, is recommended to elucidate the pathogenesis of COVID-19. Although many publications emphasize its role in the pathogenesis of SARS-CoV-2, in our study, among the markers of oxidative stress, only dimaldehyde (MDA) was found to be elevated in the serum of COVID-19 subjects. In the available literature, no potential causes of elevated levels of MDA were found. Statistical analysis was performed for lifestyle factors such as stimulant use, physical activity, and family burden, which showed no correlation of increased MDA with potential confounders/patient demographics. Clearly, there is a need for further studies evaluating this marker among patients with infections.

Reactive oxygen species (ROS) are products of cellular metabolism and are an important component of signal transduction in living organisms. Optimal levels of ROS are controlled by the antioxidant defence system, which includes enzymes and non-enzymatic antioxidants [36]. Under physiological conditions, ROS play an important role in cell signaling, the regulation of cytokine production, and growth factors, and are involved in the ageing process of the human body [37]. In certain situations, ROS levels can increase rapidly, in which case an imbalance between oxidants and antioxidant systems occurs, which is referred to as oxidative stress, leading to cell and tissue damage. Numerous publications have shown that respiratory viral infections are associated with inflammation, increased cytokine production, and other pathophysiological phenomena due to excessive ROS production [38]. Data indicating the involvement of oxidative stress in the mechanisms of initiation and the maintenance of impaired homeostasis during SARS-CoV-2 infection suggest the efficacy of antioxidant therapy in combination with viral replication inhibitors and anti-inflammatory drugs. This can be achieved by combining antioxidant and antiviral compounds or by administering antiviral drugs with compounds capable of enhancing the body’s antioxidant defences [36,39].

In a study by Pincemail et al. among COVID-19 patients hospitalized in the intensive care unit (ICU) for severe pneumonia, deficiencies in some antioxidants (vitamin C, glutathione, γ–tocopherol, β–carotene, and thiol proteins) and trace elements (selenium) were observed. Higher levels, relative to reference values, have been shown for copper and inflammatory biomarkers (CRP, myeloperoxidase) [40]. It has also been observed that the supply of nutritional doses of vitamin C, according to ESPEN guidelines, was insufficient to compensate for vitamin C deficiency in critically ill patients [41]. Only intravenous administration contributed to maintaining vitamin C concentrations within the reference range. Similarly, Zhang et al. showed that high doses of intravenous vitamin C administered for 7 days improved oxygenation in critically ill patients with COVID-19 [40,42,43,44].

Also, Mehri et al., in their study, revealed that patients with COVID-19 presented elevated levels of the following oxidative stress markers: malondialdehyde (MDA), total oxidation status (TOS), catalase (CAT), and superoxide dismutase (SOD) [45]. Respondents were further divided into ICU and non–ICU groups. It was observed that MDA levels were elevated in COVID-19 patients and correlated with disease severity as MDA levels were higher in the ICU group than in the non–ICU group. Similar results were obtained in relation to SOD. There was no significant difference between TOS and CAT levels in the ICU and non–ICU groups. However, their levels were significantly higher in the COVID-19 group compared to the control group of healthy subjects [45]. During the course of our study, a number of other publications have emerged showing elevated levels of oxidative stress markers in COVID-19 patients, highlighting their role in disease pathogenesis [46,47,48].

Thus, during the COVID-19 pandemic, it became important to search for therapies with which to effectively combat the SARS-CoV-2 virus. The results of the above studies suggest that patients with COVID-19 may benefit from strategies to reduce or prevent oxidative stress and anti-inflammatory effects.

Unfortunately, a drawback of our study is that it is not possible to compare the results of recruited patients after several months of follow-up. Based on the results of the other studies described above, we can only predict that elevated levels of IL–1Ra and IL–1β may persist for several months in patients presenting with some COVID-19 symptoms [31,32].

## Figures and Tables

**Table 1:** Demographic characteristics by study group (*p*—Mann–Whitney U test; *p* *—chi-squared test).

Parameter	Non-COVID Group	COVID Group	*p*
ABUNDANCE	24	33	
GENDER			0.516 *
F/M (%)	13/11 (45.8/54.2)	15/18 (45.5/54.5)	
Age [years]			0.625
Medium ± SD	52.21 ± 9.38	50.97 ± 9.83	
Median (min–max)	48.5 (41.0–75.0)	49.0 (35.0–86.0)	
Height [cm]			0.184
Medium ± SD	169.46 ± 8.29	172.55 ± 9.54	
Median (min–max)	170.0 (156.0–186.0)	174.0 (154.0–190.0)	
Weight [kg]			0.547
Medium ± SD	77.17 ± 14.98	78.85 ± 15.29	
Median (min–max)	77.0 (53.0–115.0)	82.0 (54.0–108.0)	
BMI [kg/m^2^]			0.917
Medium ± SD	26.85 ± 4.99	26.31 ± 3.63	
Median (min–max)	26.03 (21.19–39.84)	26.09 (19.94–34.09)	
Waist circumference [cm]			0.943
Medium ± SD	91.45 ± 10.49	90.77 ± 10.06	
Median (min–max)	94.0 (76.0–111.0)	92.0 (71.0–107.0)	
Smoking			0.208 *
YES/NO (%)	19/5 (79.2/20.8)	30/3 (90.9/9.1)	
Alcohol consumption			0.352 *
YES/NO (%)	6/18 (25.0/75.0)	5/28 (15.1/84.9)	
Physical activity			0.254 *
Low (%)	4(16.7)	8 (24.2)	
Moderate (%)	14 (58.3)	22 (66.7)	
High (%)	6 (25.0)	3 (9.1)	

**Table 2:** Selected morphological data among the study groups (*p*—Mann–Whitney U test).

Parameter	Non–COVID Group	COVID Group	*p*
AST [UL]			0.540
Medium ± SD	20.66 ± 6.05	22.4 ± 10.21	
Median (min–max)	19.0 (13.0–35.0)	21.0 (11.0–68.0)	
GGTP [U/L]			0.924
Medium ± SD	27.5 ± 26.0	51.03 ± 148.3	
Median (min–max)	22.0 (6.0–135.0)	19.0 (9.0–828.0)	
Cholesterol [mg/dL]			0.355
Medium ± SD	209.22 ± 30.72	199.5 ± 33.25	
Median (min–max)	207.11 (157.6–281.88)	204.42 (120.0–277.58)	
Cholesterol HDL [mg/dL]			0.389
Medium ± SD	52.11 ± 13.51	53.9 ± 12.53	
Median (min–max)	45.4 (31.9–80.02)	56.3 (29.13–80.7)	
Cholesterol nie–HDL [mg/dL]			0.360
Medium ± SD	156.16 ± 36.41	146.71 ± 35.45	
Median (min–max)	155.15 (77.58–241.42)	145.0 (77.74–248.45)	
Triglycerides [mg/dL]			0.008
Medium ± SD	151.64 ± 70.21	148.34 ± 237.32	
Median (min–max)	140.7 (50.43–370.6)	92.56 (43.25–1366.67)	
Creatinine [μmol/L]			0.048
Medium ± SD	66.54 ± 10.77	74.23 ± 14.53	
Median (min–max)	64.88 (48.32–89.63)	72.66 (49.21–109.07)	
Uric acid [μmol/L]			0.965
Medium ± SD	298.98 ± 64.64	313.05 ± 97.36	
Median (min–max)	296.35 (161.6–474.6)	288.9 (189.3–559.6)	
Glucose [mol/L]			0.563
Medium ± SD	5.37 ± 0.64	5.52 ± 1.83	
Median (min–max)	5.38 (4.48–7.0)	5.22 (4.48–14.92)	

**Table 3:** Cytokine levels in the blood serum of patients in the study groups. Abbreviations: IL–1Ra—Interleukin 1 antagonist; IL–1β—interleukin 1 beta (*p*—Mann–Whitney U test).

Parameter	Non-COVID Group	COVID Group	*p*
IL–1Ra			0.004
Medium ± SD	173.87 ± 73.55	276.31 ± 170.34	
Median (min–max)	166.09 (73.95–368.64)	214.72 (88.77–901.91)	
IL–1β			<0.001
Medium ± SD	5.68 ± 3.8	17.83 ± 15.47	
Median (min–max)	4.79 (1.42–15.35)	13.88 (1.51–74.76)	

**Table 4:** The levels of oxidative stress markers assessed in this study (*p*—Mann–Whitney U test).

Parameter	Non–COVID Group	COVID Group	*p*
CER mg/dL			0.179
Medium ± SD	46.49 ± 12.79	49.61 ± 14.04	
Median (min–max)	45.25 (32.27–95.11)	48.72 (16.18–85.09)	
SH µmol/L			0.812
Medium ± SD	190.9 ± 51.58	185.86 ± 58.82	
Median (min–max)	191.26 (71.09–282.27)	195.69 (0–281.65)	
SH µmol/g proteins			0.454
Medium ± SD	2.31 ± 0.6	2.2 ± 0.65	
Median (min–max)	2.31 (0.82–3.31)	2.27 (0–3.14)	
TOS µmol/L			0.153
Medium ± SD	141.81 ± 101.29	108.92 ± 92.04	
Median (min–max)	114.97 (15.3–351.7)	74.7 (12.36–323.23)	
LPH µmol/L			0.114
Medium ± SD	40.7 ± 27.48	30.25 ± 21.07	
Median (min–max)	34.66 (4.62–123.68)	23.17 (6.48–94.5)	
TAC mmol/L			0.495
Medium ± SD	1.11 ± 0.15	1.12 ± 0.18	
Median (min–max)	1.11 (0.77–1.57)	1.14 (0.7–1.48)	
SOD NU/mL			0.675
Medium ± SD	20.47 ± 1.79	20.29 ± 2.75	
Median (min–max)	20.82 (16.25–23.44)	20.4 (10.02–28.41)	
MnSOD NU/mL			0.749
Medium ± SD	10.07 ± 1.69	9.67 ± 3.48	
Median (min–max)	10.19 (7.34–13.11)	10.34 (–8.17–12.37)	
CuZnSOD NU/mL			0.749
Medium ± SD	10.39 ± 1.54	10.62 ± 2.56	
Median (min–max)	10.52 (7.83–14.25)	10.1 (7.07–18.67)	
LPS RF			0.812
Medium ± SD	350.89 ± 124.17	365.03 ± 134.06	
Median (min–max)	349.78 (152.17–655.08)	345.53 (152.17–719.41)	
MDA µmol/L			0.037
Medium ± SD	9.03 ± 4.69	6.77 ± 40	
Median (min–max)	8.2 (1.28–20.25)	6.15 (1.98–20.43)	
AGE10 [µg/mL]			0.442
Medium ± SD	782.24 ± 361.43	810.89 ± 614.21	
Median (min–max)	748.05 (226.35–1726.8)	568.65 (40.65–2311.8)	

## Data Availability

The database of aggregated statistics ready for analysis is stored in a secure and password-protected repository on the server of the Medical University of Silesia. The data were anonymized. The original contributions presented in the study are included in the article, further inquiries can be directed to the corresponding author at: gospodarczyk.alicja@gmail.com
